# The effect of anti-TNF on renal function in patients with ankylosing spondylitis: a prospective cohort study

**DOI:** 10.1007/s10067-022-06330-9

**Published:** 2022-08-12

**Authors:** I. A. P. Swart, I. M. Visman, M. Heslinga, I. E. van der Horst-Bruinsma, J. C. van Denderen, M. T. Nurmohamed

**Affiliations:** 1 Amsterdam Rheumatology and Immunology Center, Amsterdam UMC, Vrije Universiteit, Amsterdam, The Netherlands; 2 Department of Rheumatology, Amsterdam Rheumatology and Immunology Center, Jan van Breemenstraat 2, Reade, Amsterdam, 1056 AB The Netherlands

**Keywords:** Ankylosing spondylitis, Comorbidity, Epidemiology, Spondyloarthritis

## Abstract

**Background:**

Biologicals, such as anti-tumor necrosis factor (anti-TNF), reduce cardiovascular disease (CVD) in patients with inflammatory rheumatic diseases. Impaired renal function is a known predictor of CVD and elevated in ankylosing spondylitis (AS).

**Objective:**

To assess the effect of anti-TNF on renal function in patients with AS and whether anti-TNF use is safe in AS patients with pre-existing risk factors for renal decline.

**Method:**

Biological-naïve consecutive AS patients treated with etanercept or adalimumab were prospectively followed from 2005 to 2014. Renal function was determined by calculation of the estimated glomerular filtration rate (eGFR), estimated with the abbreviated modification of diet in renal disease (MDRD) formula. The effect of anti-TNF on eGFR was analyzed using mixed model analysis.

**Results:**

211 AS patients were followed for a median of 156 (36–286) weeks. Overall mixed model analyses showed a significant decrease of eGFR over time (*β* =  − 0.040, *p* = 0.000), although this association did not remain significant after adjustment for responding to anti-TNF, alcohol use, disease duration, body mass index (BMI), C-reactive protein (CRP), and disease activity (*β* =  − 0.018, *p* = 0.094). However, patients with pre-existing risk factors for renal decline did have a significant change in eGFR over time (*β* =  − 0.029, *p* = 0.006).

**Conclusions:**

We found a significant change in eGFR over time, although this small decrease was not clinically relevant. This study further demonstrates that anti-TNF does not affect renal function in AS patients with and without existing risk factors for renal decline, which means that use of anti-TNF is safe concerning renal function in patients with AS.

**Table:** 

**Key Points** *• Previous studies showed that biologicals, such as anti-tumor necrosis factor (anti-TNF), reduce cardiovascular disease (CVD) in patients with inflammatory rheumatic diseases, such as ankylosing spondylitis (AS).* *• Impaired renal function is a known predictor of CVD, and also a known concern for many AS patients.* *• Use of anti-TNF is safe with regard to renal function in patients with AS.* *• The effect of anti-TNF on CVD in AS patients does not seem to be mediated by changes in renal function.*

## Introduction

Ankylosing spondylitis (AS) is an inflammatory joint disease of the spine and pelvis. Features of AS include not only sacroiliitis, synovitis, and arthritis but also nonarticular symptoms, such as uveitis, tendinitis, and psoriasis. Patients often present with chronic back pain and are usually younger than 45 years. Commonly patients are carrier of the HLA-B27 gene [1]. The prevalence is approximately 0.7–49 per 10,000 people and depends on ethnic group, the selection of patients, and the criteria for diagnosis [2].

The most common pharmacological treatment of AS is with non-steroidal anti-inflammatory drugs (NSAIDs). Patients who do not respond to NSAIDs receive treatment with biologicals, such as anti-tumor necrosis factor (anti-TNF).

AS patients have an increased risk of cardiovascular disease (CVD) due to accelerated atherosclerosis caused by the chronic inflammatory process [3, 4]. As a result, treatment of the inflammation also leads to a decrease in atherosclerosis, which subsequently leads to reduction of CVD.

Roubille et al. [5] showed that anti-TNF reduces the incidence of CVD in other inflammatory diseases, such as rheumatoid arthritis (RA) and psoriasis. Apart from the effect via the suppression of inflammation, there is also the possibility that anti-TNF has other beneficial effects on the cardiovascular system, such as a favorable effect on renal function.

Kidney disease, caused for instance by amyloidosis, is common in AS patients [6–8]. NSAIDs are frequently used, but the downside of these drugs is their negative effect on renal function [9]. In contrast to NSAIDs, several studies report a positive effect of anti-TNF on renal function. Kim et al. [10] showed improvement in renal function with anti-TNF therapy in RA patients with chronic kidney disease. Some studies report that anti-TNF is safe in RA patients with kidney disease [11, 12]. Dönmez et al. [13] showed that anti-TNF therapy is effective in AS patients with secondary amyloidosis. However, data about the effect of anti-TNF in AS patients without kidney disease is lacking. Although some data are available in RA patients, these might not be directly extrapolated to the AS population since AS patients are younger compared to RA patients, and NSAID use is much more common [13].

Impaired kidney function is a known predictor of CVD [14]. We postulated that the favorable cardiovascular effect of anti-TNF might be mediated by the improvement of renal function.

Therefore, the aim of this study was to assess the effect of anti-TNF on renal function in patients with AS and whether anti-TNF use is safe in AS patients with beforehand existing risk factors for renal decline. If this study would show that anti-TNF is safe concerning renal function in AS patients with and without risk factors for renal decline, this might be a reason to start anti-TNF therapy earlier in patients with high cardiovascular risk and/or impaired renal function.

## Materials and methods

### Study protocol

The data for this study was collected from observational prospective cohorts: the adalimumab ASUV cohort (2005 to June 2014) and etanercept AS cohort (2005 to June 2014) of consecutive patients with AS who started anti-TNF. For this study, we used data from the 2-year follow-up period. The treatment that patients received was adalimumab subcutaneously 40 mg every other week, or etanercept subcutaneous 50 mg per week or 25 mg twice a week. The treating rheumatologist was responsible for the treatment allocation. Several variables were assessed at baseline and after 4, 12, 24, 52, 78, and 104 weeks of therapy. The ethics committee approved this study and all patients gave written informed consent.

### Patients

Patients were included in this study if they were diagnosed with AS and fulfilled the modified New York criteria (1984) [15]. They had to qualify for starting anti-TNF treatment; this means a Bath Ankylosing Spondylitis Disease Activity Index (BASDAI) more than 4 and failure or inadequate response to more than two NSAIDs [16]. For this study, patients were required to have baseline assessment and at least one follow-up assessment including renal function. Patients were excluded if they had contraindications to anti-TNF or if they used biologicals in the past.

### Baseline characteristics

Gender, age, ethnicity, smoking, alcohol consumption, disease activity, disease duration, history of CVD, history of uveitis, HLA-B27 positivity, medication use, body mass index (BMI), waist-hip ratio, blood pressure, erythrocyte sedimentation rate (ESR), C-reactive protein (CRP), and serum creatinine were assessed.

### Renal function

Renal function was determined by calculation of the estimated glomerular filtration rate (eGFR) using the abbreviated modification of diet in renal disease (MDRD) formula for glomerular filtration rate (GFR) in ml/min/1.73 m^2^, as this is currently the most used formula for estimating kidney function. The MDRD formula: 32,788 * (serum creatinine in μmol/l^(−1.154)^) * (age in years^(−0.203)^) * 0.742 if female and/or * 1.212 if ethnic black [17].

### Risk factors

We investigated patients with different risk groups for renal decline. Factors considered as risk factors were the following: eGFR < 60 ml/min/1.73 m^2^, NSAID use, excessive alcohol consumption, age > 60 years, systolic blood pressure > 140 mmHg, and smoking. Alcohol consumption was considered excessive upward of 14 units per week for men and 7 units per week for women. Response to anti-TNF was defined as either a 50% improvement in the BASDAI score at 3 months or a BASDAI improvement at 3 months of 2 points or more [18].

### Statistical analysis

Baseline characteristics were presented as mean and standard deviation (SD) for continuous variables if normally distributed, median and interquartile range (IQR) for continuous variables if not normally distributed, or percentage (%) for frequencies.

Descriptive techniques were used to present data over time. The effect of anti-TNF on eGFR was analyzed using longitudinal analysis techniques (mixed model analysis), using time of anti-TNF use as independent variable and eGFR as dependent variable. A *p*-value of < 0.05 was considered significant. Analyses were adjusted for possible confounders. All baseline variables were regarded as potential confounders and were considered as actual confounders if the regression coefficient altered with a minimum of 10%. All analyses were performed using SPSS V.23.0.

## Results

### Patients

**Fig. 1:**
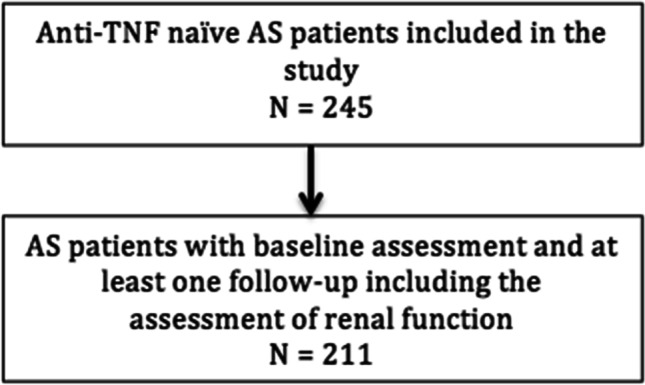
Flowchart of patient selection

In total, 211 consecutive patients were included in this study. The median follow-up time was 156 (36–286) weeks. Figure 1 shows the flowchart of patient selection for this study.

### Baseline characteristics

Baseline characteristics are shown in Table 1 for the total study population.

**Table 1:** Characteristics of AS patients

	All patients (*n* = 211)
Demographics	
Men (%)	67.3
Age (years)	42.56 ± 11.24
Ethnicity, Caucasian (%)	73.2%
Lifestyle	
Current smokers (%)	40.6%
Alcohol consumption (units/week)	1.00 (0.00–4.75)
Disease related	
BASDAI	5.85 ± 1.89
ASDAS-CRP	3.48 ± 0.97
Disease duration (years)	8.00 (2.00–14.00)
History of CVD (%)	1.9
History of uveitis (%)	18.0
HLA-B27 positive (%)	80.8
Use of medication	
NSAID use (%)	79.6
Antihypertensives use (%)	16.7
Statin use (%)	10.0
DMARD use, without prednisone (%)	21.0
Prednisone use (%)	2.9
Physical examination	
BMI (kg/m^2^)	26.53 ± 7.09
Waist-hip ratio (cm/cm)	0.89 ± 0.09
Systolic blood pressure (mmHg)	127.94 ± 17.21
Laboratory	
ESR (mm/h)	18 (7.00–34.00)
CRP (mg/L)	7 (3.00–20.00)
Serum creatinine (μmol/l)	73.11 ± 17.73
eGFR, MDRD formula (ml/min/1.73^2^)	104.94 ± 24.20

Results are presented as mean and standard deviation (SD), median and interquartile range (IQR) or percentage (%)

*BASDAI*, Bath Ankylosing Spondylitis Disease Activity Index; *ASDAS-CRP*, Ankylosing Spondylitis Disease Activity Score using CRP; *NSAID*, non-steroidal anti-inflammatory drugs; *DMARD*, disease modifying anti-rheumatic drugs; *BMI*, body mass index; *ESR*, erythrocyte sedimentation rate; *CRP*, C-reactive protein; *eGFR*, estimated glomerular filtration rate

### Association between anti-TNF use and renal function in patients with AS

**Fig. 2:**
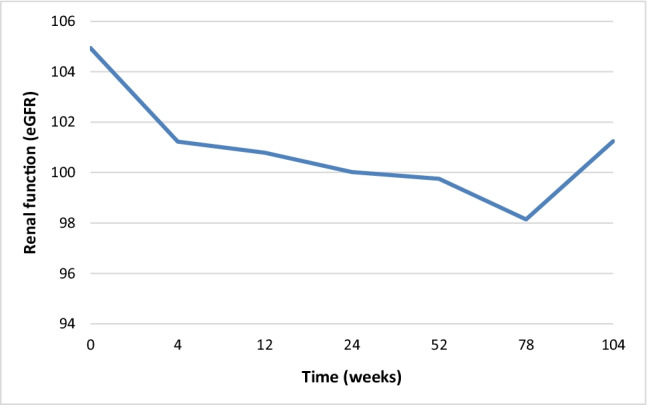
eGFR on average over time after starting anti-TNF in AS patients

Renal function decreased over time (Fig. 2). EGFR at baseline was 104.94 (SD = 24.20) ml/min/1.73 m^2^, which decreased to 99.75 (SD = 22.40) ml/min/1.73 m^2^ after 1 year of treatment (delta − 3.9, SD = 16.9) and to 101.24 (SD = 20.64) ml/min/1.73 m^2^ after 2 years of treatment (delta − 2.4, SD = 15.6). For responders, the decrease at 1 year was − 4.2 (SD = 15.5), and − 3.3 (SD = 14.5) at 2 years.

In the crude mixed model analysis, we found that the use of anti-TNF was associated with decrease in renal function (*β* =  − 0.040, *p* = 0.000). However, after adjustment for responding to anti-TNF, alcohol consumption, disease duration, BMI, CRP, and disease activity (BASDAI), the effect did not remain significant (adjusted *β* =  − 0.018, *p* = 0.094) (Table 2).

**Table 2:** Mixed model analyses for the effect of anti-TNF use on renal function in AS patients

Model	Estimate	95% CI Lower bound	95% CI Upper bound	*p*-value
Crude	− 0.040	− 0.063	− 0.019	0.000
Adjusted*	− 0.018	− 0.040	0.003	0.094
≥ 1 risk factor*	− 0.029	− 0.049	− 0.008	0.006

^*^Model is adjusted for responders, alcohol, disease duration, BMI, CRP, and disease activity (BASDAI)

### Association between anti-TNF use and renal function in patients with AS and have one or more risk factors for renal decline

However for patients (*n* = 196, 93%) who had one or more risk factors for renal decline, we found a significant decrease in renal function over time (*β* =  − 0.029, *p* = 0.006) (Table 3). For patients with one risk factor (*n* = 97, 46%), we found only a significant decrease in renal function over time for patients with the risk factor age > 60 year (*β* =  − 0.060, *p* = 0.016). We found no significant change over time for patients who had only one risk factor which were the following: eGFR < 60 ml/min/1.73 m^2^ (*β* = 0.0112, *p* = 0.058), NSAID use (*β* =  − 0.020, *p* = 0.063), men with excessive alcohol consumption (*β* =  − 0.015, *p* = 0.692), women with excessive alcohol consumption (*β* = 0.047, *p* = 0.191), systolic blood pressure > 140 mmHg (*β* = 0.023, *p* = 0.442), or smoking (*β* =  − 0.019, *p* = 0.190).

**Table 3:** Mixed model analyses for the effect of anti-TNF use in AS patients with only one already existing risk factor for renal

Model	Estimate	95% CI Lower bound	95% CI Upper bound	*p*-value
eGFR < 60	− 0.112	− 0.229	0.004	0.058
NSAID use	− 0.020	− 0.042	0.001	0.063
Excessive alcohol consumption (men)	− 0.015	− 0.091	0.061	0.692
Excessive alcohol consumption (women)	0.047	− 0.025	0.118	0.191
Elderly (> 60 year)	− 0.060	− 0.108	− 0.012	0.016
Systolic blood pressure > 140 mmHg	0.023	− 0.037	0.083	0.442
Smoking	− 0.019	− 0.047	0.009	0.190

## Discussion

To our knowledge, this is the first prospective study to assess the effect of anti-TNF on renal function in AS patients without kidney disease. In contrast to the findings of previous studies, which showed improvement of renal function in AS and RA patients with kidney disease, we did not find such an association between anti-TNF and renal function alteration in AS patients [10, 13]. In contrast, we did find a small decrease in renal function in AS patients that disappeared after adjustment for alcohol consumption, disease duration, BMI, CRP, and disease activity (BASDAI). Furthermore, for patients with one or more risk factors for renal decline, we found a significant (mean − 3.9, SD 16.7), but not clinically relevant (i.e., not a sustained decrease in eGFR of 15 ml/min/1.73 m^2^ or more per year [17]), decrease in renal function over time. We found a very small but not clinically relevant change of eGFR in all patients. The decrease might be explained by the patients’ increase of age over time, since eGFR decreases when patients grow older, e.g., Qin et al. found a yearly decrease in men without kidney disease of − 1.9 per year [19]. Our findings are in line with the previous studies concerning the safety of anti-TNF on renal function [10–13].

Important strengths of our study are the prospective data collection and the longer period of time that patients were followed with many visiting moments. There were many detailed assessments, which involved all clinical relevant values. The data for this study was collected from cohorts: the adalimumab ASUV cohort (2005 to June 2014) and etanercept AS cohort (2005 to June 2014), which are real-life cohorts of consecutive patients with AS. Since we had much detailed data available, we were able to investigate if the effect of anti-TNF on renal function was different for patients with risk factors for renal decline, which were not investigated in previous studies. The real-life cohorts and investigation of AS patients with risk factors for renal decline imply that the results of this study are generalizable to the larger AS population.

There are several limitations in this study. First, only patients with normal renal function were followed. As there were only a few people with impaired renal function, these results are not generalizable to the population with renal impairment. As a result, the results of this study are not directly comparable with previous studies. Second, there were some missing data, although these data were missing completely at random (MCAR). This means that a relevant bias is not likely. Furthermore, the use of mixed model analysis also minimizes the impact of the missing data on the results [20]. Last, to estimate renal function, we calculated eGFR by means of serum creatinine, which is less precise compared to direct measurements of eGFR or calculation of eGFR using serum cystatin C. Another limitation is that we did not use a control group of patients with AS not on anti-TNF therapy. It would be interesting for a future study to compare AS patients with and without anti-TNF in comparison to the population.

This study demonstrates that anti-TNF does not affect renal function in AS patients with and without beforehand existing risk factors for renal decline, which means that use of anti-TNF is safe concerning renal function in patients with AS. From our results, it seems that the effect of anti-TNF on CVD in AS patients is not mediated by an effect on renal function. Future research might unravel if and how the favorable effect of anti-TNF on CVD is mediated by other variables than renal function.

## Data Availability

The authors have full control of all primary data and agree to allow the journal to review their data if requested.
